# Editorial: Unveiling the host’s acute immune response to infectious mucosal diseases: insights and implications

**DOI:** 10.3389/fimmu.2025.1672088

**Published:** 2025-08-15

**Authors:** Mohd Arish, Carmen Fernández, Farha Naz

**Affiliations:** ^1^ Beirne B. Carter Center for Immunology Research, University of Virginia, Charlottesville, VA, United States; ^2^ Department of Molecular Biosciences, The Wenner-Gren Institute (MBW), Stockholm University, Stockholm, Sweden; ^3^ Department of Medicine, Division of Infectious Diseases and International Health, University of Virginia, Charlottesville, VA, United States

**Keywords:** infection - immunology, *Salmonella Typhi* (*S Typhi*), TLR4, TLR5, macrophages, RBC (red-blood-cell), TLR9, LPS

Innate immunity constitutes the body’s first line of defense, acting with remarkable specificity and speed in response to microbial threats. Once considered a blunt instrument of host defense, the innate immune system is now recognized as highly nuanced, capable of immunological memory, developmental crosstalk, and tissue-specific modulation (1, 2). The contributions in this Research Topic reflect the growing appreciation of innate immune complexity across diverse systems and life stages, from bacterial infection to tissue repair and early-life development.

The study by Toapanta et al. employs a Controlled Human Infection Model (CHIM) with *Salmonella Typhi* to reveal distinct alterations in monocyte subsets during infection. Classical and intermediate monocytes in individuals who reached typhoid diagnosis criteria (TD) upregulated pattern recognition receptors (TLR4, TLR5), phagocytic markers (CD36, CD206), and gut-homing integrins (α4β7). These findings resonate with prior studies showing monocytes as dynamic responders capable of migrating to mucosal tissues and differentiating into effector macrophages (3, 4). The observed expansion of activated CM clusters suggests that monocytes may act not only as precursors to intestinal macrophages but also as immune amplifiers during systemic infection, shaping both innate and adaptive responses (5).

Extending the role of non-traditional immune cells, Xiao et al. present compelling evidence that red blood cells (RBCs), long considered immunologically inert, express surface TLR9 capable of binding mitochondrial DNA (mtDNA). Their data show that in bacterial infections, the number of mtDNA bound to RBCs increases significantly and correlates with C-reactive protein (CRP) levels, a marker of systemic inflammation. This aligns with emerging views that extracellular mtDNA is a potent damage-associated molecular pattern (DAMP) capable of triggering TLR9 and cGAS-STING pathways (6, 7). The discovery that RBCs may act as immune sentinels through TLR9 expands their functional repertoire and opens new avenues for biomarker development in infectious diseases.

In contrast to these inflammation-driven responses, Soma et al. propose a beneficial immune modulation model via mucosal administration of lipopolysaccride (LPS). Traditionally viewed as an endotoxin that drives sepsis when administered systemically (8), LPS can have markedly different effects when delivered orally or transdermally. The authors introduce the “macrophage network,” a framework wherein environmental LPS primes mucosal macrophages, which in turn communicate with distal tissue-resident macrophages through juxtacrine signaling. This hypothesis resonates with prior findings that low-dose LPS exposure can induce endotoxin tolerance and protective effects (9, 10). Their review article reframes LPS not as a uniform danger signal but as a context-dependent modulator of immune tone and tissue homeostasis.

Complementing these findings, Sharafian et al. utilize infant-derived ileal enteroids to explore how innate cytokines shape epithelial maturation. Their model reveals that IL-22, secreted by neonatal Th17 cells, drives epithelial proliferation and secretory differentiation while downregulating Wnt and Notch pathways. These results support a growing body of literature positioning IL-22 as a central regulator of mucosal barrier integrity and antimicrobial defense (Sharafian et al., 11, 12). The findings also reinforce the developmental specificity of immune–epithelial crosstalk, with early-life cytokines serving dual roles in tissue formation and immune readiness.

Together, these studies underscore the functional plasticity of the innate immune system. From circulating monocytes and epithelial crosstalk to erythrocyte surveillance and macrophage conditioning, innate immunity emerges as a finely tuned network capable of integrating microbial, developmental, and environmental signals. These insights challenge traditional compartmentalizations of immune cell function and suggest that innate cells operate not just as pathogen destroyers but as orchestrators of homeostasis, repair, and long-term immunity (13).

We thank all contributors to this Research Topic for their high-quality work. Their findings not only expand the functional map of innate immunity but also offer translational potential from infection biomarkers and vaccine design to mucosal therapies and early-life interventions. As the field continues to move beyond static classifications, future studies will benefit from high-resolution tools such as single-cell transcriptomics, spatial mapping, and *in vivo* imaging to further decode the cellular choreography of innate immunity in health and disease.
